# Human Monocyte Subsets and Phenotypes in Major Chronic Inflammatory Diseases

**DOI:** 10.3389/fimmu.2019.02035

**Published:** 2019-08-30

**Authors:** Theodore S. Kapellos, Lorenzo Bonaguro, Ioanna Gemünd, Nico Reusch, Adem Saglam, Emily R. Hinkley, Joachim L. Schultze

**Affiliations:** ^1^Genomics and Immunoregulation, Life and Medical Sciences Institute (LIMES), Bonn, Germany; ^2^Platform for Single Cell Genomics and Epigenomics, German Center for Neurodegenerative Diseases and University of Bonn, Bonn, Germany

**Keywords:** human monocytes, atherosclerosis, diet, respiratory diseases, neurodegeneration

## Abstract

Human monocytes are divided in three major populations; classical (CD14^+^CD16^−^), non-classical (CD14^dim^CD16^+^), and intermediate (CD14^+^CD16^+^). Each of these subsets is distinguished from each other by the expression of distinct surface markers and by their functions in homeostasis and disease. In this review, we discuss the most up-to-date phenotypic classification of human monocytes that has been greatly aided by the application of novel single-cell transcriptomic and mass cytometry technologies. Furthermore, we shed light on the role of these plastic immune cells in already recognized and emerging human chronic diseases, such as obesity, atherosclerosis, chronic obstructive pulmonary disease, lung fibrosis, lung cancer, and Alzheimer's disease. Our aim is to provide an insight into the contribution of human monocytes to the progression of these diseases and highlight their candidacy as potential therapeutic cell targets.

## Introduction

Human monocytes were originally defined by their distinctive morphology at the beginning of the previous century by Paul Ehrlich and Ilya Metchnikoff [reviewed in (1)]. The invention of flow cytometry in the 1970s enabled the design of a monocyte-specific antibody panel based on the surface protein levels of the pattern recognition receptor CD14 and the Fc gamma III receptor CD16 (2).

Two populations were identified; the classical (CD14^++^CD16^+^) and the non-classical (CD14^dim^CD16^+^) (2). Subsequently, an intermediate for CD14 and CD16 (CD14^+^CD16^+^HLA-DR^+^CD86^+^CD11c^+^) monocyte population with a distinct transcriptomic profile (*LYZ, S100A8, CD14, S100A10, HLA-DRA, CD74, IFI30, HLA-DPB1, CPV*) was discovered (3–5). At this time, it was also suggested that this population can be separated from non-classical monocytes by the expression of 6-sulfo LacNAc (SLAN) (6). These “intermediate” monocytes displayed comparable ROS production and phagocytosis potential, lower adhesion to surfaces, but demonstrated higher class II molecule expression and IL-12 production than classical monocytes (3, 4). In mice, two monocyte subsets were identified in the bloodstream by flow cytometry and intravital microscopy; a short-lived Gr-1^+^CCR2^+^CX_3_CR1^lo^ which migrates to tissues during inflammation and a Gr-1^−^CCR2^−^CX_3_CR1^hi^ one, which carries out CX_3_CR1-dependent patroling of the vasculature during homeostasis (7–9).

Investigation in the developmental trajectories of the three described monocyte subsets with deuterium labeling in humans suggested that intermediate and non-classical monocytes emerge sequentially from the pool of classical monocytes (10). In fact, mathematical modeling of monocyte differentiation demonstrated a linear trajectory from classical monocytes to non-classical monocytes, although it is very likely that not all of them follow the same path or that the intermediate to non-classical monocyte step occurs outside the bloodstream (11). An elegant study provided another line of evidence to support this concept. In particular, endotoxin challenge led to a rapid loss of all monocyte subsets. However, their re-appearance from the bone marrow or marginated pools followed different kinetic patterns; classical monocyte numbers were restored first, with intermediate and non-classical monocytes following. Of note, the first two subsets followed a peak in CCL2, CCL3, and CCL4 blood levels, in contrast to the latter which were sensitive to CX_3_CL1 (12). In mice, monocyte development clearly occurs in the bone marrow where granulocyte-monocyte (GMP) and monocyte-DC (MDC) progenitor pools produce functional monocytes (13). Furthermore, during infections, monocyte progenitor reprogramming happens already in the bone marrow (14).

With the development of multi-dimensional single-cell techniques, assessment at the single-cell transcriptome level unexpectedly suggested 4 monocyte subsets in healthy volunteers; classical, non-classical, and 2 monocyte subsets, one expressing genes involved in cell cycle, differentiation and trafficking and the other being associated with a NK cell-like signature (15). By generating new single-cell transcriptomics data we now have evidence that the latter monocyte subset was due to misclassification of a particular subset of NK cells, indicating that the current model with 3 major subsets is still valid (16).

Classical monocytes were found to be primed for phagocytosis, innate sensing/immune responses and migration, intermediate monocytes were the only subset expressing *CCR5* and were well-suited for antigen presentation, cytokine secretion, apoptosis regulation, and differentiation and non-classical monocytes are involved in complement and Fc gamma-mediated phagocytosis and adhesion (17, 18). However, it was also concluded that the current monocyte subsets are not homogeneous populations, but instead can be clustered in smaller, transcriptionally distinct subsets (17).

Using a mass cytometry approach, Thomas and colleagues showed that traditional gating on CD14 and CD16 frequently led to contaminations of intermediate and non-classical monocytes; instead, the addition of markers, such as CD36, CCR2, HLA-DR, and CD11c enabled more precise separation of human monocytes (19). Another mass cytometry protocol increased the resolution of the non-classical monocyte phenotype and distinguished CD14^dim^CD16^+^SLAN^−^ from CD14^dim^CD16^+^SLAN^+^ non-classical monocytes. All non-classical monocytes in this study exhibited less CD36, CD64, CCR2, CD11b, and CD33, but more CD45, CD11c, and HLA-DR expression than classical and intermediate monocytes, becoming consistent in terms of useful surface marker selection for reliable monocyte subset isolation (20). Lastly, another study counted 8 monocyte clusters in healthy individuals using a broad range of lineage, adhesion, antigen presentation, migration, activation, cell death, and survival markers. Classical monocyte subsets differed on the levels of IgE, CD61/CD9, and CD93/CD11a, whilst non-classical monocyte subsets were further divided by the expression of CD9 and SLAN which linked them to increased efferocytosis and migration to CCL16 in comparison with SLAN^−^ non-classical monocytes (21). It will be interesting to learn in larger cohorts of healthy and diseased individuals whether such cellular subsets are of functional relevance *in vivo*.

Monocyte subsets have been shown to exhibit distinct functional properties which partly rely on differential methylation status of immune-related genes (22). For example, classical monocytes migrate to CCL2 and CCL3 gradients and are more efficient than intermediate monocytes in producing ROS and constraining fungi (23–25). In fact, CD14^+^ human monocytes express higher levels of chemokine receptors, such as CCR1, CCR2, CCR5, CXCR1, and CXCR2 which highlights their potential to migrate to cues stemming from injured or inflamed tissues (18, 24), but are also characterized by their ability to secrete pro-inflammatory molecules, such as IL-6, IL-8, CCL2, CCL3, and CCL5 (18, 26). Based on evidence originating from murine studies [reviewed in (1, 27)], but also recent human observations (28), it is now widely accepted that classical monocytes have the ability to differentiate into monocyte-derived macrophages (moMϕs) and DCs (moDCs) (29) and play an integral part in shaping inflammation and its resolution in tissues.

Intermediate monocytes express the highest levels of antigen presentation-related molecules (18, 30) and were also shown to secrete TNF-α, IL-1β, IL-6, and CCL3 upon TLR stimulation (18, 26, 31), while Szaflarska and colleagues described an anti-tumoral phenotype for these cells (32). With regard to chemokine receptors, they express more CCR5 than classical monocytes and this likely accounts for their high susceptibility to HIV-1 infection (5, 24, 33). CD14^+^CD16^+^ monocyte numbers are expanded in the blood of patients with systemic infections, implying that they must play an important role in the rapid defense against pathogens (34, 35). However, their exact role in immunity remains elusive as another report found that they are the main producers of IL-10 upon TLR stimulation (36). Whether these cells can produce pro- and anti-inflammatory mediators simultaneously or whether there are different kinetics of expression for these factors requires further exploration.

On the other hand, a comparison of CD16^+^ and CD16^−^ monocytes revealed that despite the remarkable similarities which suggest a common developmental origin, CD16^+^ cells possess a more mature phenotype -as assessed by transcriptome profiling- and associate with gene ontology terms, such as cell-to-cell adhesion, cell trafficking, proliferation, and differentiation (37). In addition, they express higher levels of CX_3_CR1 which explains the fact that they migrate and adhere more than CD16^−^ monocytes to fractalkine-secreting endothelium (5, 25).

Non-classical human monocytes express a distinct transcriptomic and metabolic profile (respiratory chain metabolism) in comparison to classical monocytes which utilize carbohydrate metabolism as their energy source (38). Similar to CD14^+^CD16^+^ monocytes, they present antigen processing capabilities, but are distinguished from classical monocytes by their association with wound healing processes (38). Furthermore, they have antagonizing functions to classical monocytes and promote neutrophil adhesion at the endothelial interface via the secretion of TNF-α (39) and do not reach the classical monocyte production levels of pro-inflammatory cytokines (40). Finally, a role for the SLAN^+^ subset of non-classical monocytes in TNF overproduction in viraemic HIV-infected patients was proposed, suggesting that they might be considered as a major actor in the immune hyperactivation of the disease (41). While SLAN seems to delineate a subset of non-classical monocytes, there is no evidence for transcriptional differences between SLAN^+^ and SLAN^−^ cells (16) which requires further work to understand the reasons for the discrepancy between homogeneity at the transcriptional, but heterogeneity at the protein level.

In recent years the concept of trained immunity has been introduced (42). Monocytes exposed to ß-glucan or BCG react toward a related secondary stimulus with a faster onset and a more pronounced inflammatory response (43–45). Surprisingly, it is not entirely clear, whether all monocyte subsets can exert such a response or whether only a subset of monocytes is capable to be programmed in such a way. Furthermore, it is unclear whether there are changes in the training response when monocytes transition from classical via intermediate to non-classical monocytes.

**Figure 1:**
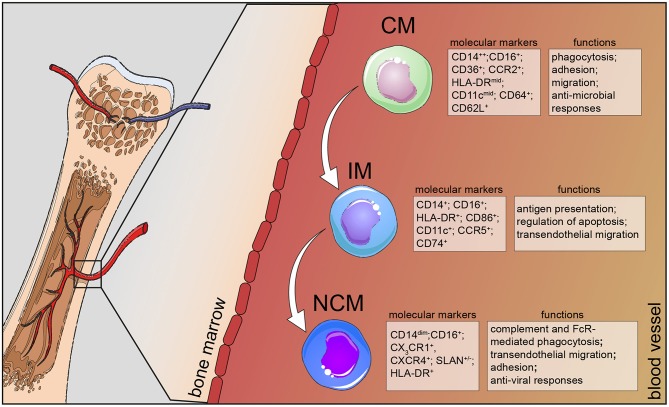
Human monocyte subsets in health. Human monocytes mature in the bone marrow and are subsequently released into the circulation as CD14^+^ classical monocytes. Progressively, classical monocytes (CD14^+^CD16^−^) give rise to non-classical monocytes (CD14^dim^CD16^+^) through an intermediate step of CD14^+^CD16^+^ monocytes. Classical monocytes in humans can be distinguished from the other two subsets by additional markers, such as CD36, CCR2, and CD64 and take part in the host's anti-microbial responses, such as adhesion to the endothelium, migration, and phagocytosis. Intermediate monocytes are characterized by their high expression of CCR5 and HLA-DR molecules and are involved in antigen processing and presentation and transendothelial migration. Non-classical monocytes divide into a SLAN^+^ and a SLAN^−^ population, express high levels of CX_3_CR1 and specialize in complement and FcR-mediated phagocytosis, transendothelial migration and anti-viral responses. CM, classical monocytes; IM, intermediate monocytes; NCM, non-classical monocytes.

**Figure 2:**
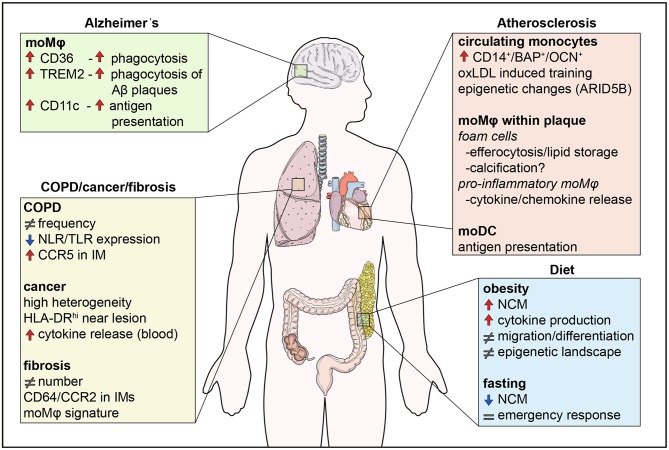
Monocyte functions in disease. Monocytes are involved in human diseases both by their direct functional effects, but also indirectly through their differentiation into macrophages. Diet influences the numbers of non-classical monocytes, monocyte migration, and cytokine production, effects which are counteracted by fasting. In addition, the epigenetic landscape is altered by metabolites in a process called innate immune memory. In atherosclerosis, monocytes differentiate into foam cells which secrete pro-inflammatory cytokines and chemokines, store lipids and are possibly involved in calcification. Differentiation of monocytes to DCs also contributes to antigen presentation. In the lung, changes in monocyte numbers are the most common observation in disease. Monocytes display high heterogeneity and their functions may be impaired like in COPD, whereas monocyte location seems to be crucial in lung cancer, with monocytes close to tumors being immunocompromised. Finally, monocytes infiltrate the brain in neurodegenerative diseases, such as Alzheimer's disease. CD36 and TREM2 are upregulated and enhance phagocytosis of Aβ plaques in monocyte-derived macrophages. NCM, non-classical monocyte; IM, interstitial macrophage.

Taken together, human monocyte subsets display remarkable heterogeneity in their surface marker expression and function; classical monocytes exhibit a more pro-inflammatory phenotype via their ability to secrete soluble mediators and to differentiate into monocyte-derived DCs to bridge innate and adaptive immune responses, intermediate monocytes are specialized in antigen presentation and play an important role in HIV infections, while non-classical monocytes are responsible for the anti-viral responses of this lineage (Figure 1). In this review, we summarize the most recent findings on monocyte behavior in human chronic diseases and put extra emphasis on phenotypic changes that occur and correlate with disease severity or progression. We decided to focus on chronic inflammatory diseases, such as atherosclerosis, diet-induced syndromes, respiratory diseases, and neurodegenerative conditions as case studies for the heterogeneity and plasticity that these cells exhibit in humans (Figure 2).

## Major Lifestyle Changes and Their Effects on Human Monocytes

Non-communicable diseases (NCDs) are among the main causes of death in western countries. The close correlation between dietary habits and morbidity and mortality of chronic NCDs has been already extensively characterized (46, 47). In the last 20 years the shift to a more sedentary lifestyle and a Western type high-caloric diet has led to a continuously growing percentage of obese and overweight people (48). According to the World Health Organization, more than half the European population is overweight, of which 30% is obese (49), with this percentage also dramatically increasing in South America and Asia (50). Additionally, obesity is clearly associated with an increased risk of several co-morbidities, such as type 2 diabetes (T2D), cardiovascular disease (CVD), atherosclerosis, kidney and liver failure, sterile inflammation (51, 52), and certain types of cancer (53).

Dietary habits have been shown to drastically affect the number and composition of the three populations of circulating monocytes. Obesity has been shown to induce monocytosis of the intermediate and non-classical subsets (54, 55), while transcriptomic analysis of monocytes in obese donors demonstrated increased expression of *TLR4* and *TLR8* and secretion of pro-inflammatory cytokines, such as IL-1β and TNF in response to LPS or ssRNA stimulation (54).

Moreover, all circulating monocytes in obese donors express more CX_3_CR1, implying an increased chemotactic potential toward CX_3_CL1-secreting adipocytes (54). In accordance with this observation, obesity has been characterized by an increased amount of monocyte-derived adipose tissue macrophages in both mouse and human (53, 56). Caloric restriction has beneficial effects in many chronic metabolic disorders like T2D, non-alcoholic liver disease and CVD (57–59) and short-term fasting is sufficient to reduce the numbers of all monocyte populations in healthy human subjects (60).

Nevertheless, in depth characterization of mechanistic changes occurring due to different dietary habits is still lacking. Modern high-dimensional technologies (e.g., multi-color flow cytometry, mass cytometry, single-cell RNA-seq) will contribute to understanding primary and secondary effects of diet on the monocyte compartment, possibly dissecting the influence of single macronutrients.

## Human Monocyte Differentiation in the Gut is Influenced by Dietary Components

Metabolites play a major role in the differentiation of monocytes and affect their functionalities, as exemplified by the short chain fatty acid β-hydroxybutyrate, which upon its release from the liver under prolonged fasting, has been shown to suppress the NLRP3 inflammasome-induced IL-1β and IL-18 production by human monocytes (61). Similarly, Goudot and colleagues found that *in vitro* activation of human monocytes with 6-formylin-dolo(3,2-b)carbazole (FICZ), an endogenous ligand for the environmental sensor aryl hydrocarbon receptor (62), biases monocyte differentiation into moDCs via a BLIMP-1-dependent mechanism (63). Finally, bacterial butyrate imprints a host protection program via epigenetic remodeling during monocyte to macrophage differentiation in the lamina propria (64). In more detail, in the absence of tissue-damaging inflammation, butyrate induces macrophages to upregulate antimicrobial proteins, such as calprotectin.

The mechanisms by which metabolites alter monocyte functions have many aspects in common with the concept of innate immune cell memory where initial priming with a stimulus leads to sustained epigenetic reprogramming that culminates in a phenotypic change upon subsequent challenge (42, 65). Emerging evidence on diet-associated triggers shows that they can induce cellular reprogramming in humans. For instance, *in vitro* exposure of human monocytes to oxidized low density lipoprotein (oxLDL) reprograms the cells to enhance the expression of pro-inflammatory cytokines and chemokines (66). Furthermore, single nucleotide polymorphisms at the gene regions of the inflammasome adaptor ASC and the IL-1 receptor antagonist were identified to have an effect on the training response of human monocytes to oxLDL which proposes the involvement of the inflammasome in this process (67). Taken together, diet-related triggers may induce differential levels of training in human individuals, thus adding another layer of heterogeneity to human monocyte immune responses.

## Monocytes and Monocyte-Derived Cells in Atherosclerosis

Atherosclerosis is triggered -at least in part- by the elevated levels of oxLDL and LDL which accumulate in the intima of arterial walls (68, 69). A vicious cycle of infiltrated immune cells which store lipid species in the intima and recruit more leukocytes leads to the formation of atherosclerotic plaques, mostly situated in branching points of the vessels. The consequences of ruptured plaques and subsequent clogging of arteries include myocardial infarction and stroke which are the leading causes of death worldwide (70).

Monocytes play a key role in the early formation and maturation of plaques. They are attracted to the arteries by chemokines, such as CCL2 secreted by activated endothelial cells (71–77) and take up lipids within the subendothelial space to differentiate into foam cells (76, 78). Additionally, they can phagocytose precipitated cholesterol crystals (79) and oxidized lipid species (66, 80, 81) that activate the inflammasome, leading to a highly inflammatory form of cell death called pyroptosis and the induction of innate immune responses (79).

Research on the functional differences of human monocytes in atherosclerosis provided mechanistic insight into their role in the disease. Isolation of monocytes from individuals with symptomatic coronary atherosclerosis and elevated levels of the CVD risk factor lipoprotein(a) displayed a long-lasting pro-inflammatory phenotype (80, 82, 83). These functional differences are accompanied by changes in the monocyte epigenetic landscape. For example, the expression of pro-inflammatory genes such, as *TNF, IL6, CCL2*, and *CD36* in oxLDL-trained monocytes are regulated by trimethylation of H3K4 residues at the promoter regions (66). Similarly, a large study in control and CVD patients showed that the expression of the transcription coactivator ARID5B positively correlates with CVD. It acts by removing repressive H3K9me2 histone marks from its target genes which are related to inflammatory/immune responses, chemotaxis, extravasation, and phagocytosis (84).

To date, epidemiological studies investigating the correlation between circulating monocytes and the occurrence of cardiovascular events or atherosclerosis severity using flow cytometry have yielded contrasting results due to technical and experimental design reasons (74, 85–96). Briefly, Hamers and colleagues first showed that SLAN^+^CXCR6^+^ non-classical monocytes are more frequent in patients with atherosclerosis. This subset presents with a higher capacity to migrate toward the chemokine CXCL16 secreted by macrophages in plaques and is probably involved in the clearance of apoptotic cells from the necrotic core (21). On the other hand, a longitudinal study on a larger cohort revealed a correlation of classical monocytes with reverse cardiac events and a negative association of intermediate monocyte numbers with plaque thickness (96). The correlation of elevated monocyte counts and higher risk for cardiac events has been confirmed in other reports, as well (90). However, classical monocyte counts could not be associated with plaque stability or increased chance of cardiac events after carotid endarterectomy in patients with already existing atherosclerotic plaques (94). Additionally, other studies demonstrated that elevated intermediate monocyte counts play a pivotal role in the growth and stability of already existing atherosclerotic plaques or cardiac attacks (86–88, 97, 98). Elevated CCL2 levels in the early phases of the development of atherosclerotic plaques may lead to increased classical monocyte counts and could thus be considered a predictive marker, while later on, the presence of necrotic cores may rather recruit non-classical monocytes to control vascular homeostasis and the clearance of debris (21, 99, 100).

Computational deconvolution of whole transcriptome data from 126 human carotid plaques using known signatures for leukocytes revealed that macrophages represent about 50% of cells in human atherosclerotic plaques (101). The origin of these cells in human atherosclerotic plaques is not fully resolved. Lin and colleagues argued for the monocyte origin of all described macrophage subtypes in murine atherosclerotic plaques by lineage tracing of bone marrow-derived myeloid cells (102). Interestingly however, only a proportion of the foam cells exhibits a monocyte origin (103) which is compatible with the concept of smooth muscle cell transdifferentiation into foam cells and macrophage-like cells (104–107).

The role and heterogeneity of monocyte-derived cells in the atherosclerotic plaques needs to be further investigated as they could serve as therapeutic targets. As a matter of fact, single-cell RNA-seq analysis of atherosclerotic plaques provided an insight into the heterogeneity of macrophages present in transgenic mouse models. Three main subsets of macrophages have been identified; the resident-like macrophages, which probably overlap with aortic resident macrophages present in steady state, a set of pro-inflammatory macrophages and a subtype of macrophages with a high expression of *Trem2* and genes associated with lipid-metabolic pathways and cholesterol efflux (101, 108). As shown by Kim and colleagues, the *Trem2*^hi^ cells probably reflect the lipid-laden foam cells (103). The most diverse macrophage spectrum in atherosclerotic plaques was so far presented in mice (102), with some of the conserved markers also being validated in human atherosclerotic plaques (101, 108). Furthermore, all single-cell RNA-seq studies of the murine plaque environment defined subsets of DCs. Whereas Kim and colleagues subdivided DC subsets into DC1 and DC2 (103), Cochain et al. found only one DC subset which they hypothesized to be monocyte-derived (108). Indeed, monocyte lineage tracing also included a subset of DC-like cells which was termed as CD74^hi^MHC^hi^ macrophages (102). These results indicate that at least part of the DCs found in atherosclerotic plaques may be of monocyte origin. Interestingly, the moDCs of the latter study differentially express *Ahr* which has been associated with monocyte to DC differentiation and they are more present during plaque progression rather than regression (63, 108).

Monocyte-derived cells may also contribute to the calcification of the cap, another major feature of the atherosclerotic plaque, despite the earlier consensus that it is mainly mediated by the transdifferentiation of smooth muscle cells to osteoclast-like cells (109). Single-cell RNA-seq of murine plaques revealed a macrophage subset expressing osteoclast genes like osteopontin and human plaques express their protein products (108). Notably, there is a unique osteogenic monocyte subtype in humans defined by the expression of CD14^+^, bone alkaline phosphatase and osteocalcin which is linked to the degree of calcification and the burden of necrotic cores (110). These cells may also differentiate into calcium-depositing macrophages upon transmigration.

In summary, the composition and ontogeny of monocyte-derived cells in the atherosclerotic plaques has been well-described in mice. Monocytes are recruited to the intima of arteries upon lipid deposition and differentiate into a spectrum of pro-inflammatory macrophages, lipid-laden foam cells, and DCs. Experimental limitations still hamper the translation of these findings to humans. In addition, the contribution of different monocyte subsets to disease progression suffers from low temporal and functional resolution in epidemiological studies. A focus on high-dimensional phenotyping of plaque-associated macrophages, monocytes and their progenitors in humans will allow a deeper understanding of disease development and will hopefully lead to novel therapeutic targets.

## Monocyte Phenotype and Functions in Human Respiratory Diseases

At steady state, the myeloid compartment of the human lung consists of the CD163^+/++^CD206^+^CD64^+^CD14^lo^ alveolar macrophage population, CD169^−^ interstitial macrophages, CD14^+^ tissue monocytes and two populations of (CD1a^+/−^) monocyte-derived cells (111–113). Monocytes express typical blood monocyte markers, such as CD14, CD11b, CCR2, and CD16, but at extravascular sites they possess higher levels of CD141, CD11c, HLA-DR, and CCR7, indicating a tissue-imprinted phenotypic change that is reminiscent of DCs (111, 114). Indeed, location is key for monocyte functions, as exemplified by the enrichment of intermediate monocytes in distal airways and the weaker production of pro-inflammatory mediators than in the peripheral blood (114). Similarly, accumulation of CD141^+^CD14^+^ pulmonary mononuclear phagocytes at the T cell zones of draining lung lymph nodes likely facilitates antigen presentation and T cell-mediated immunity (111).

Changes in monocyte counts have been observed in muco-obstructive lung diseases and fibrotic disorders (115, 116). For example, total numbers of monocytes and the non-classical subset change in the blood of patients with chronic obstructive pulmonary disease (COPD) (117), while classical monocyte counts can be a prognostic marker of mortality in patients with idiopathic pulmonary disease (IPF) (118). However, other monocyte subsets may contribute to disease progression, as shown for intermediate monocytes expressing CD64 or CCR2 (119). Finally, monocytes do not only affect disease outcome by their direct functions, but also through their differentiation to macrophages. This was shown in IPF patients where alveolar macrophages expressed a gene signature similar to that of monocyte-derived macrophages of bleomycin-treated animals (120).

Studies using bulk transcriptomics suggested that monocytes express a shared gene signature with alveolar macrophages which is overexpressed in COPD compared to healthy individuals and correlates with lung function (121). However, these studies are hampered by the fact that immune cells are treated as homogeneous populations and thus a direct link between peripheral blood monocyte subsets with distinct phenotypes and alveolar macrophage populations in the bronchi of patients with COPD is missing. To resolve this issue, it will be necessary to employ single-cell technologies, such as single-cell RNA-seq and study the differentiation trajectories of peripheral blood monocytes and lung-derived myeloid populations.

Moreover, the frequency of CD206^+^ non-classical monocytes is reduced, while that of CD163^+^CD206^+^CCR5^+^ is increased (117). Congruent with this, intermediate monocytes from patients with COPD also overexpressed CCR5 as a result of high systemic IL-6 and sIL-6R levels. However, their migration capability to CCL5 or CXCR3 chemokines in comparison with non-smokers was reported to be either impaired (122) or not affected at all (123). Lastly, miRNAs could account for monocyte functional dysregulation. Dang and colleagues showed that increased miR-24-3p expression in blood T cells and monocytes from patients with COPD was associated with decreased levels of genes involved in the TLR and NLR pathways which remains to be experimentally validated (124).

Lung cancer is one of the most prevalent cancers worldwide (125) and non-small cell lung cancer (NSCLC) is the most common subtype. Although T cells have been extensively studied in the past, the importance of monocytes in the disease is starting to emerge as recent evidence links their levels to a greater risk of recurrence (126) and worsened post-operative disease-free and overall survival rate (127, 128). The power of single-cell transcriptomics to deconvolute the immune cell structure of NSCLC was evaluated both in mouse and humans (129). Lung tissue-derived myeloid cells in NSCLC patients were divided into 14 transcriptional states; three populations carried signatures of monocytes, 9 of macrophages, one of monocytes/DCs and one subset was characterized by cell cycle ontology terms. In alignment with human monocyte subset expression signatures, the lung monocyte populations identified in this study were defined as classical (*CD14, FCN1*), non-classical (*CDKN1C, LILRB2, ITGAL*), and neutrophil-like (*S100A8, S100A9, CSF3R*). Nevertheless, although these monocyte populations matched to the three major peripheral blood monocyte populations from the same study, on average, considerable transcriptional differences were seen, such as those related to expressed chemokines and chemokine receptors (129).

Similar to the findings in COPD, tumor infiltrating CD14^+^ cells in patients with early lung cancer express a mixture of FcγRs (CD64, CD32), cytokine receptors (CD115) and scavenger receptors (CD163, CD206). Further phenotyping revealed that monocyte/TAM localization is driven by microenvironmental cues and thus HLA-DR^hi^ TAMs are found at the tumor lesion, whereas HLA-DR^lo/−^ monocytes reside at distant sites (130). Of note, tumor monocytes displayed a compromised ability to stimulate T cells in direct contrast to TAMs. These results are in line with a previous report on stage I lung adenocarcinoma which found that CD14^+^ and CD16^+^ monocyte numbers are decreased at the tumor site, express less HLA-DR than macrophages and secreted less IL-8 and IL-1β at the tumor site compared to monocytes at the rest of the tissue (131). With single-cell-omics technologies now entering this field, we anticipate further knowledge about spatial and temporal changes of monocytes in blood, lung parenchyma, and bronchoalveolar lavage in these major lung diseases.

## Human Monocytes in Neurodegenerative Diseases: Case Study in Alzheimer's Disease

Neurodegenerative diseases are disorders that disturb the proper functioning of neurons in the central nervous system (CNS). They may affect the structure or the survival of the neurons, which are unable to regenerate after the damage, thus leading to cognitive or motor dysfunction. The immune system was only recently found to play an important role in neuronal injury that occurs in an inflammatory milieu through a complex interplay between resident (microglia) and infiltrating myeloid cells (monocytes) (132, 133). The major neurodegenerative diseases include Alzheimer's disease (AD) which affects over 150 million people worldwide (134, 135), Parkinson's disease (136), Huntington's disease (137), and amyotrophic lateral sclerosis (138).

AD is characterized by the accumulation of insoluble amyloid beta (Aβ) in the extracellular matrix which forms plaques and of hyperphosphorylated tau protein in the cytoplasm which forms neurofibrillary tangles (139). Studies have shown that these protein aggregates are strongly associated with neuroinflammation, synaptic loss and impaired neuronal function which ultimately lead to cognitive decline (135, 140, 141). The progressive deposition and aggregation of Aβ peptides in the brain are the result of an imbalance between their production and clearance, a process in which brain-resident microglia and brain-infiltrating peripheral monocytes (moMϕs) are involved (134, 142).

Because of the technical and ethical limitations of human CNS studies, most of the work on the molecular mechanisms of neurodegenerative diseases has been conducted in murine disease models. The infiltration of monocytes in the brain through the blood-brain barrier was shown in murine models to be mediated via the CCL2-CCR2 axis with microglia and recruited monocytes located in the close proximity of deposited Aβ plaques (143), although some controversial studies based on irradiation experiments exist (144). In the context of monocyte infiltration to the brain, monocytes differentiate into moMϕs which upregulate the expression of surface proteins, such as CD11c, TREM2, and CD36 (145). In this study, Martin and colleagues sorted microglia as (CD45^mid^CD11b^+^) and moMϕs as (CD45^hi^CD11b^+^), a common strategy also used in other studies (146–148). The role of monocytes in AD is multi-faceted. In a larger consortium led by the Neher group, we recently provided evidence that a systemic immune response to LPS stimulation can lead to localized immune training in the brain (149). This, alongside the knowledge that AD is often accompanied by a systemic inflammatory response (150), poses the question: how does the interaction behind this bidirectional relationship work? Whether inflammation is a result of AD pathology or not, an earlier causal factor or both of these at once is yet to be answered.

A human study using blood and brain tissue from healthy and old subjects found that increased expression of the myeloid cell surface receptor CD33 is linked to the AD risk allele rs3865444C (151). This is noteworthy as it expands previous work in murine models whereby increases in CD33 expression lead to higher uptake of Aβ42 peptides and lower deposition of Aβ plaques (152). Building on this, another study confirmed the relationship between CD33 and risk allele rs3865444C, further suggesting that it can result in higher surface expression of TREM2, another biomarker of AD pathology in the cortex (153).

Recently, there has been a renewed interest in the link between TREM2 expression and AD pathology, in particular where the late onset forms are concerned (154). In the presence of functional TREM2, CD68-positive microglial activity initially promotes the clearance of Aβ aggregates by triggering microglia clustering around the plaques. However, due to the concomitant overexpression of ApoE levels in the vicinity of the plaques, Aβ deposition is progressively enhanced. On the other hand, TREM2 loss-of-function mutant mice reported higher levels of Aβ seeding, suggesting TREM2 involvement is a double-edged sword.

The characterization of AD in the brain has recently been advanced by emerging single-cell technologies which allow an in-depth look at changes in aging transcriptomes. One study assessed age-related microglia changes by examining gene expression profiles of purified parietal cortex microglia leading to the identification of human-specific signatures. The study suggests that with increasing age, microglia downregulate actin cytoskeleton-related genes (*TLN1, PFN1, EVL, ARPC1A, ARPC1B, CORO1A, CAP1, CTNNA2, VASP*) and cell surface receptors (*P2RY12, IL6R, TLR10*) (155). More recently, a dataset consisting of 80,660 single nuclei transcriptomes from AD patients' human prefrontal cortexes at different stages of the disease indicated the existence of heterogeneity in six identified cell types. Four microglial subpopulations were identified and *CD81, SPP1, APOC1, PTPRG*, and *APOE* were highly upregulated in AD samples. In addition, these subpopulation profiles uncover new AD-associated genes, including the complement component *C1QB* and *CD14*, which have not been reported before. Interestingly, transcriptional changes in response to the earlier disease stages were more cell type-specific in comparison with more ubiquitous late stage variations where the genes being upregulated represented a more general stress response (156).

In none of these single-cell sequencing studies on AD presented above have researchers been able to identify bone marrow-derived monocytes. The reason for this could be monocyte exclusion in sorting panels, such as in Galatro et al. (155) or the utilization of known marker genes in cell type classification as in Mathys et al. (156). In contrast, single-cell studies on pre-clinical models of other neuroinflammatory diseases, such as multiple sclerosis, the numbers of microglia and circulating monocytes in the brain have been shown to be increased in comparison with homeostasis (157). Consequently, the way monocytes are involved in neurodegenerative diseases depends on both the condition itself and the severity stage.

## Closing Remarks

Human monocytes are still widely studied in context of peripheral blood and the advent of novel single-cell technologies, including sequencing-based methods have fueled new interest in these cells. While higher heterogeneity has been suggested, we still propose classical, intermediate, and non-classical monocytes as the three major subsets within the monocyte cell space. We would suggest further heterogeneity being explained by functional states of these important immune cells. However, this requires a community effort with guidelines on how to define such newly defined cell states in the monocyte compartment. This will also be important in view of the increasing interest in tissue-associated monocytes and their ability to differentiate into moMϕs or moDCs. Of particular interest are current and future studies on spatio-temporal behaviors of monocyte-derived cells within diseased tissues and organs. We are convinced that the new single-cell technologies can help to decipher the role of these important cells during the major chronic, but also acute inflammatory diseases.

## Author Contributions

All authors listed have made a substantial, direct and intellectual contribution to the work, and approved it for publication.

### Conflict of Interest Statement

The authors declare that the research was conducted in the absence of any commercial or financial relationships that could be construed as a potential conflict of interest.

## Abbreviations

AD: Alzheimer's disease
Aβ: amyloid beta
CNS: central nervous system
COPD: chronic obstructive pulmonary disease
CVD: cardiovascular disease
IPF: idiopathic pulmonary fibrosis
moDC: monocyte-derived dendritic cell
moMΦ: monocyte-derived macrophage
NCD: non-communicable disease
oxLDL: oxidized low density lipoprotein
SLAN: 6-sulfo LacNAc
T2D: type 2 diabetes
IM: interstitial macrophage.
